# Sandwich-like transparent ceramic demonstrates ultraviolet and visible broadband downconversion luminescence†

**DOI:** 10.1039/c8ra02195c

**Published:** 2018-04-10

**Authors:** Yue Hu, Zheng Li, Wei Pan

**Affiliations:** State Key Laboratory of New Ceramics and Fine Processing, School of Materials Science and Engineering, Tsinghua University Beijing 100084 P. R. China panw@mail.tsinghua.edu.cn

## Abstract

We report a novel sandwich-like Ce, Yb:Y_1.76_La_0.18_Zr_0.06_O_*x*_ transparent ceramic, which shows efficient UV/Vis to NIR downconversion luminescence with a broad conversion window (250 nm to 650 nm), owing to its graded defective structure. The solar spectrum conversion efficiency is improved by 3.6 fold, compared with the homogenous material.

## Abstract 2

A novel sandwich-like transparent ceramic with a graded defective structure shows efficient downconversion luminescence with a broad conversion window.

## Graphical abstract

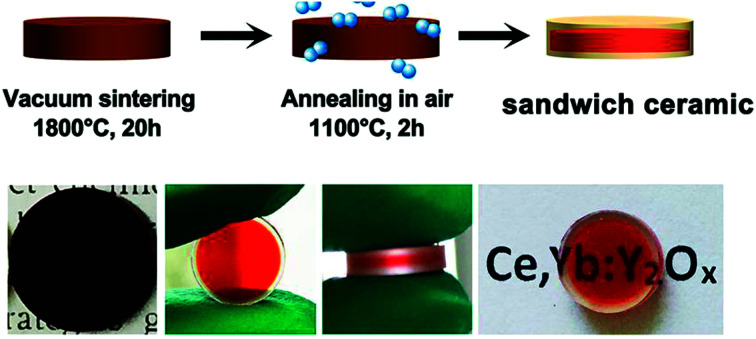

The major obstacle limiting the efficiency of crystalline silicon (c-Si) solar cells is the mismatch between the spectral response curve of c-Si and the solar spectrum distribution.^1^ The bandgap energy (*E*_g_) of c-Si is approximately 1.1 eV. Sunlight photons of energy greater than the *E*_g_ cannot be absorbed efficiently by c-Si, with extra energy being wasted as heat losses, therefore the external quantum efficiency cannot be higher than the Shockley–Queisser limit (∼30%).^1^ One way in which to break this limit is by using downconversion materials to convert the short wavelength part of the solar spectrum into wavelengths closer to *E*_g_ before it reaches the solar cell.^2,3^ Previous efforts have shown that Re–Yb (Re = Tb, Tm, Pr or Ce) doped downconversion materials are promising materials for application in spectrum modified solar cells, which can convert high-energy UV/Vis photons into more (∼1000 nm) photons through Re → Yb cooperative energy transfer.^4–10^ However, the narrow, discrete, line-like conversion band of Tb^3+^/Tm^3+^/Pr^3+^ ions, due to their forbidden f–f transition, is inefficient for harvesting of the broadband solar spectrum.^11,12^ On the other hand, Ce ions demonstrate a broader absorption window (400–600 nm) owing to the allowed Ce 4f → 5d transition,^13–17^ but this absorption range is still not wide enough for application in sunlight harvesting.

Yttria based transparent ceramic has been an attractive optical material in the past decade because it shows high transparency (98% of theoretical transmittance),^18^ a wide transmittance range (250–2500 nm) and good chemical/thermal stability.^19^ These advantages make it a promising material for use in spectrum modified solar cells, either as a luminescent down-shifting layer (LDS) on the top surface of solar cells,^20^ or as a luminescent solar concentrator (LSC) with solar cells coupled on its edges.^21^ Its good transparency in a large transmittance range indicates that photons with a wavelength beyond the conversion window can transmit through the spectral converter without optical losses, therefore common absorption of photovoltic materials such as c-Si will not be harmed.^11^ In addition, the emerging transparent ceramic technology also makes it easy to fabricate novel optical materials with complex structures, such as gradient-doping ceramic composites or cladding-core configuration fibres.^22^

Here, we designed a novel sandwich-like Ce, Yb co-doped Y_1.76_La_0.18_Zr_0.06_O_*x*_ (YLZO) transparent ceramic composite with a graded-defective structure. The introduction of defects improves the light absorption effectively, and the broadband downconversion luminescence with a large conversion window covering the whole UV-Vis region (250–650 nm) is realized for the first time. The sandwich ceramic also shows good transparency, which is necessary for avoiding the common absorption decrease of photovoltic materials, while being used as a spectral converter for solar cells.

The sandwich-like transparent ceramic was prepared by vacuum sintering and a tailored air-annealing process (experimental details are shown in the ESI†). In a typical routine (Fig. 1), the cold-isostatic-pressed disk type green body of raw powders was vacuum sintered at 1800 °C for 20 h under 1.0 × 10^−3^ Pa, and a dark brown color Ce, Yb:YLZO ceramic was obtained. The dark color of the ceramic is due to the high concentration of oxygen defects that come from high temperature sintering under a vacuum atmosphere, which is very common in transparent ceramic sintering.^23–25^ In transparent ceramic fabrication, this is usually followed with long-term annealing at a relative high-temperature under an air atmosphere to eliminate the oxygen defects.^22,26^ During the air annealing, the following defect reaction will occur:1
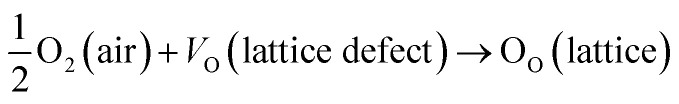


**Fig. 1:**
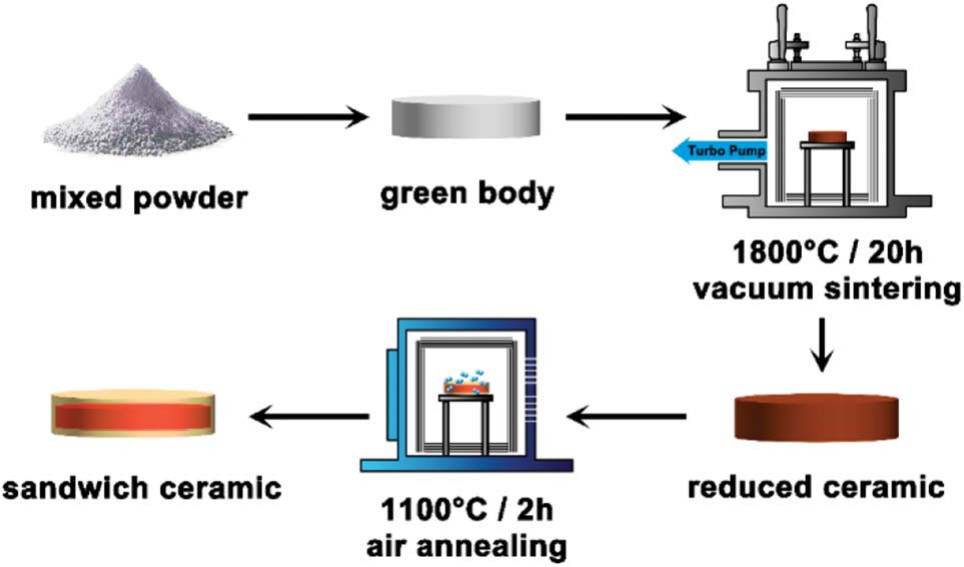
Synthesis procedure of the sandwich-like Ce, Yb:YLZO transparent ceramic.

In which, *V*_O_ indicates the oxygen vacancy, and O_O_ is the oxygen at the lattice site. The limiting step of reaction (1) is generally oxygen diffusion in the solid ceramics at annealing temperatures. In this work, we controlled the annealing temperature and the time taken to partially eliminate the oxygen defects and obtain a sandwich-like oxygen defect graded distribution transparent ceramic as shown in Fig. 1 and 2a. From Fig. 2a it can be seen that when the vacuum-sintered ceramic disk is annealed at 1100 °C under an air atmosphere for 2 h, the color reduces due to the diffusion of oxygen from air into the highly oxygen defective ceramic bulk, and a graded yellow/red/yellow colored sandwich-like Ce, Yb:YLZO ceramic is obtained.

**Fig. 2:**
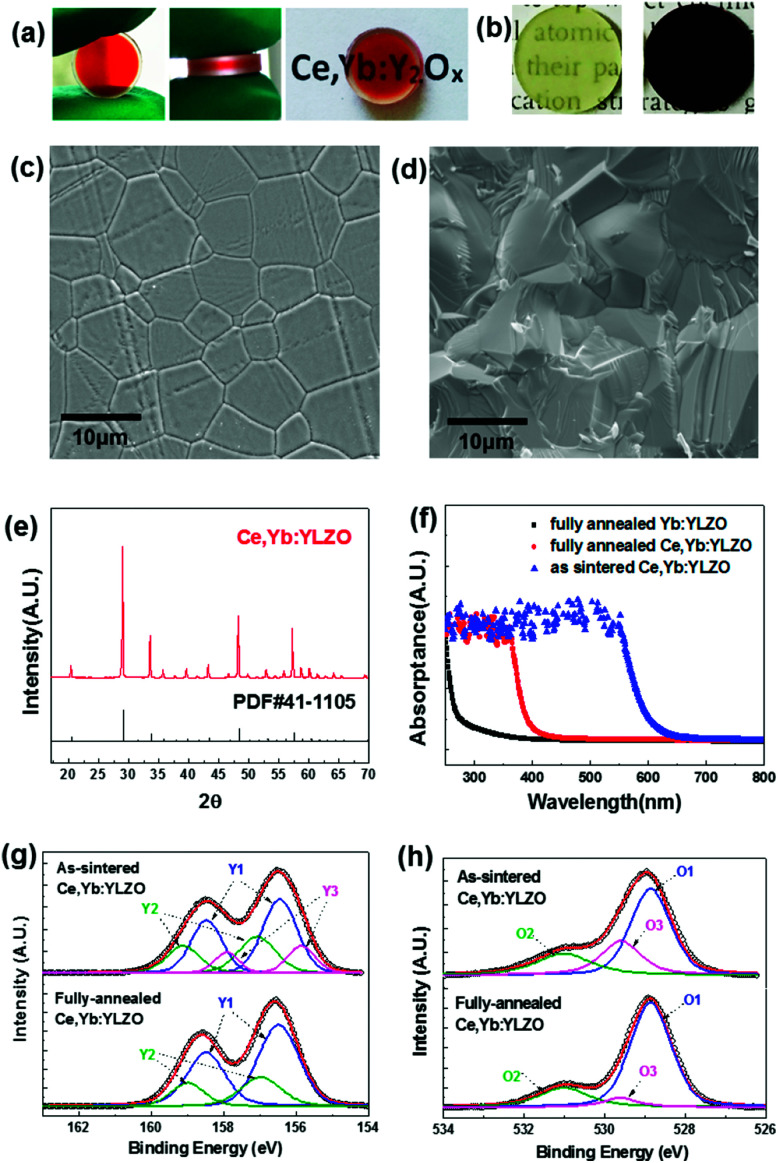
(a) Photos of the sandwich-like Ce, Yb:YLZO ceramic. (b) Photos of the fully-annealed (yellow) and as-sintered (dark brown) Ce, Yb:YLZO ceramic. (c) and (d) SEM images. (e) XRD plots. (f) UV-Vis absorption spectra. (g) and (h) XPS spectra of Y 3d and O 1s. Y1: Y–O bond (156.5 and 158.5 eV), Y2: Y–OH bond (157.0 and 159.0 eV), Y3: Y_2_O_3−*x*_ (155.8 and 157.9 eV), O1: lattice oxygen (528.9 eV), O_2_: absorbed oxygen (531 eV) and O3: oxygen defects (529.6 eV).

Fig. 2a and b also show that the ceramic is fully densified and transparent. The top and bottom layers are light yellow, owing to the doping of Ce ions and a low oxygen defect concentration.^14^ The color of the mid-layer is red, probably due to the light absorption of color centers caused by the high concentration of the oxygen defect.^22,23^ In Fig. 2b, the color of the as-sintered ceramic changed from dark brown to a homogeneous light yellow when it was annealed at 1450 °C for 10 h under an air atmosphere.

The SEM photos (Fig. 2c and d) show that the sandwich-like ceramic is a fully densified polycrystalline with a grain size around 10 μm. The XRD pattern (Fig. 2e) agrees well with the cubic Y_2_O_3_ phase (JCPDS 41-1105), and no second phases are observed in the XRD results. Fig. 2f shows the UV-Vis absorption spectra measured for the as-sintered Ce, Yb:YLZO, fully-annealed Ce, Yb:YLZO and fully-annealed Yb:YLZO ceramics. UV absorption (250–400 nm) is clearly observed in the fully-annealed Ce, Yb:YLZO ceramic, which can be attributed to the combination effect of the host lattice absorption,^27^ Yb–O charge transfer absorption^28^ and Ce 4f → 5d absorption.^29^ The absorption window of the as-sintered Ce, Yb:YLZO ceramic extends into the visible region (<650 nm), indicating that the Vis light absorption can be enhanced by introducing oxygen defects. XPS results show that the Y 3d peaks of the as-sintered Ce, Yb:YLZO splits into three doublets corresponding to the Y–O bond,^30^ the Y–OH bond,^31^ and the Y_2_O_3−*x*_.^32,33^ After fully annealing in air, the Y_2_O_3−*x*_ peak vanishes, indicating that the oxygen defect concentration is lower than the XPS detection limit (Fig. 2g). For the O 1s de-convoluted spectrum, there is an obvious oxygen defect peak (529.6 eV) in the as-sintered Ce, Yb:YLZO,^34,35^ which decreases greatly after air annealing owing to the defect elimination during oxygen diffusion into the highly defective Ce, Yb:YLZO (Fig. 2h).

Photoluminescence (PL) and photoluminescence excitation (PLE) spectra measurements were conducted at room temperature to study the downconversion properties (Fig. 3). It can be seen that the as-sintered, fully-annealed, and sandwich-like Ce, Yb:YLZO ceramics are all efficient downconversion materials. The PL emission peaks in the NIR region (Fig. 3c) are located at 976, 1030 and 1074 nm, which can be attributed to the Yb: ^2^F_5/2_ → ^2^F_7/2_ transition.^36^ The peak positions of different specimens are consistent, which indicates that the introduction of oxygen defects does not change the position of the Yb 4f energy levels. It can be seen that the 976 nm emission peak decreases greatly in the sandwich Ce, Yb:YLZO ceramic under visible wavelength excitation. This is due to the reabsorption effect of the Yb ions,^37,38^ which is detrimental to the downconversion efficiency, and we will aim to eliminate this in future work.

**Fig. 3:**
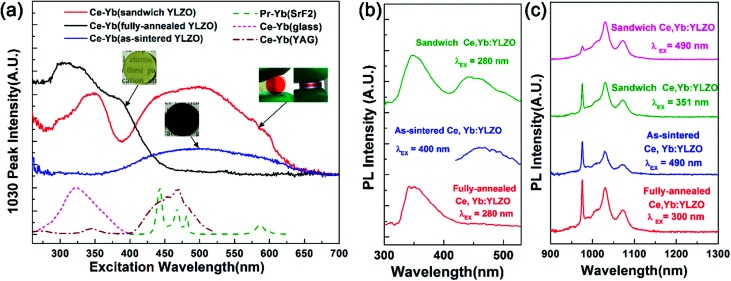
(a) PLE spectra measured with the fully-annealed, as-sintered and sandwich-like Ce, Yb:YLZO transparent ceramics (monitoring emission peak 1030 nm). The PLE spectra of Ce, Yb:glass, Ce, Yb:YAG and Pr, Yb:SrF_2_ are also listed for comparison. (b) and (c) PL spectra measured with fully-annealed, as-sintered and sandwich-like Ce, Yb:YLZO transparent ceramics.

Fig. 3b shows the PL spectra of the as-sintered, fully-annealed, and sandwich-like Ce, Yb:YLZO ceramic in the visible region. It can be seen that the fully-annealed Ce, Yb:YLZO ceramic shows ∼350 nm emission under 280 nm excitation, which is similar to that in Ce:Lu_2_O_3_ (ref. 39) and can be attributed to the Ce 5d → 4f emission. In the PL spectra of the sandwich Ce, Yb:YLZO ceramic, there is another broadband emission at around 450 nm, which is also found in the as-sintered Ce, Yb:YLZO ceramic under 400 nm excitation. This emission is caused by the introduction of oxygen defects, which indicates that there is probably a defect-induced mid-gap state under the Ce 5d level.

Fig. 3a shows that the effective excitation band of the fully-annealed Ce, Yb:YLZO ceramic is located at 250–450 nm, which can be attributed to the Ce 4f → 5d transition.^14^ The excitation band of the as-sintered Ce, Yb:YLZO ceramic is located at 400–650 nm owing to the defect-induced red-shift effect. A typical Ce^3+^ transition band is not seen in the PLE spectra of the as-sintered Ce, Yb:YLZO ceramic, because the oxygen-defect-induced color centers can cause luminescence quenching in the short wavelength region where the Ce^3+^ transition is located.^23,40,41^ Downconversion caused by the Yb–O charge transfer band has been observed in many host materials.^42,43^ However, it is not seen in this work, because the Yb–O charge transfer band decays quickly in the bulk Y_2_O_3_ host (lifetime ∼ 72 ns) and quenches easily with increasing temperature (quenching temperature ∼ 130 K).^28^

In the PLE spectra of the sandwich-like Ce, Yb:YLZO ceramic, excitation bands in both the UV and Vis range (250–650 nm) have been detected (Fig. 3a, red line). The UV excitation band of the sandwich-like Ce, Yb:YLZO ceramic is similar to that of the fully-annealed Ce, Yb:YLZO ceramic, which can be attributed to the Ce 4f → 5d transition. The excitation peak position changes from approximately 300 nm to approximately 350 nm, probably due to the change of crystal field around the Ce^3+^ ions in the gradient defective structure. On the other hand, the Vis excitation band of the sandwich-like ceramic is similar to that of the as-sintered Ce, Yb:YLZO ceramic, indicating that the Vis to NIR downconversion of the sandwich-like ceramic contributes to the highly oxygen defective layer. These results confirm that the graded sandwich-like Ce, Yb:YLZO ceramic is a broadband (UV and Vis) downconversion material combining the conversion ability of both the fully-annealed and as-sintered Ce, Yb:YLZO. The conversion range is broader than that of any known downconversion material, such as Ce–Yb doped glass, Ce–Yb doped crystals or Pr–Yb doped materials.^14,44^ Which demonstrates that the sandwich-like Ce, Yb:YLZO ceramic has a remarkable broadband spectral conversion ability converting photons in the whole UV-Vis region into approximately 1000 nm photons.

Previous efforts have confirmed that there are a large amount of oxygen vacancies in vacuum sintered transparent ceramics.^23,24^ First-principle calculation suggests that the introduction of oxygen vacancies can induce a mid-gap state in yttria-base materials (Fig. S1 and S2†), which is probably the reason why the conversion band of the highly defective Ce, Yb:YLZO red-shifts from the UV to the Vis region. As shown in Fig. 4a, the usual Ce → Yb downconversion process contains three steps.^14^ Firstly, Ce ions absorb UV photons and jump to the excitation state. Secondly, energy transfers from the Ce ions to the Yb ions, pumping Yb ions to the excitation state. Thirdly, Yb ions jump back to the ground state, emitting photons at approximately 1000 nm. In highly oxygen defective materials, the absorption red-shifts to the Vis region, pumping Ce ions into a defect-induced mid-gap state (Fig. 4b). The following two steps stay the same, and the UV → NIR downconversion process becomes a Vis → NIR downconversion process. PL and PLE results suggest that this mid-gap level locates at about 2.5–2.8 eV above the 4f level of Ce ions, which agrees with the result calculated by the first principle theory (2.7–2.9 eV).^45^ These results suggest a new strategy to adjust the spectral conversion window of the Ce–Yb downconversion material through defect tuning, which may help in the future development of spectral conversion materials and spectral modified photovoltaic devices.

**Fig. 4:**
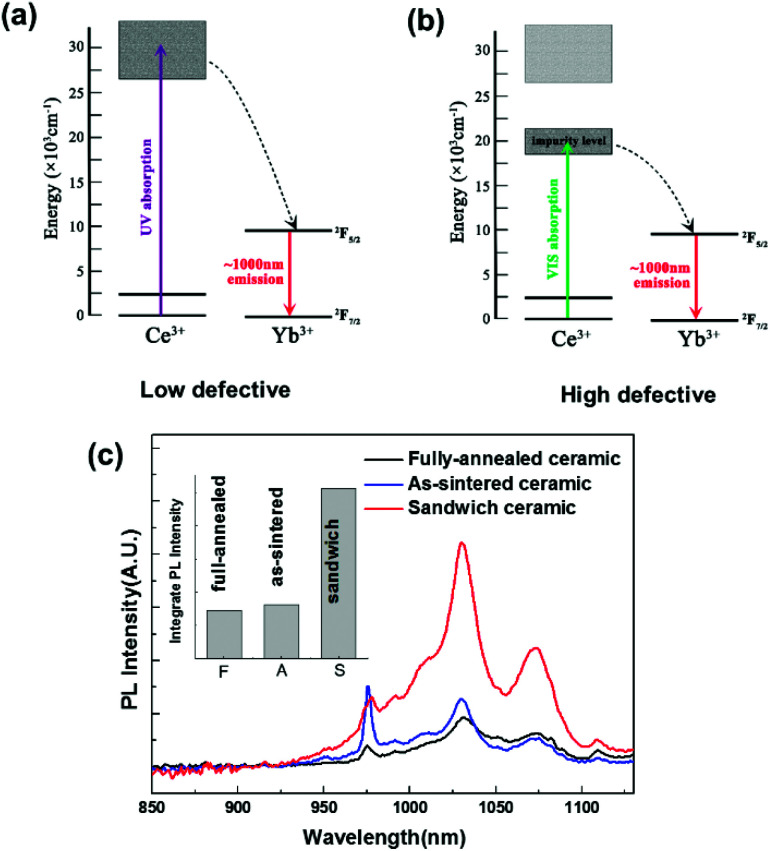
(a) and (b) Energy transfer diagram of Ce → Yb downconversion luminescence process. (c) PL spectra using a solar simulator as the excitation source (inset: integrated luminescent intensity).

For practical applications, downconversion materials with a broad excitation bandwidth are of significant advantage because more photons in the solar spectrum can be converted effectively to the desired wavelength.^11^ To investigate the solar-spectrum conversion ability, the PL spectra of the fully-annealed, as-sintered and sandwich-like Ce, Yb:YLZO ceramic was measured under the illumination of a solar simulator. As shown in Fig. 4c, the solar-excited PL intensity of the sandwich-like Ce, Yb:YLZO ceramic is significantly stronger than that of the fully-annealed ceramic or the as-sintered ceramic, owing to its broader conversion window. In general, the integrated PL intensity is proportional to the spectral conversion efficiency.^13^ According to the result shown in the inset of Fig. 4c, the spectral conversion efficiency of the sandwich-like ceramic is 3.6 times larger than that of its homogenous counterpart, which demonstrates that designing a graded defective structure is an effective way to improve the efficiency of spectral conversion materials.

In conclusion, we have developed a new strategy to tune the excitation band of Ce–Yb downconversion materials with oxygen defects, and broadband downconversion has been realized by preparing a low defect/high defect/low defect sandwich-like transparent ceramic with a controlled air-annealing treatment after vacuum sintering. This unique structure effectively extends the spectral conversion window and improves the solar conversion efficiency by 3.6 times, which may help to enhance the performance of spectral modified photovoltaic devices.

## Conflicts of interest

There are no conflicts to declare.

## Supplementary Material

RA-008-C8RA02195C-s001
