# Supplementary material for: Sandwich-like transparent ceramic demonstrates ultraviolet and visible broadband downconversion luminescence
Source: RSC Adv. 2018 Apr 10;8(24):13200–4. doi: 10.1039/c8ra02195c (PMC9079731; doi:10.1039/c8ra02195c)
Supplement: RA-008-C8RA02195C-s001 [file RA-008-C8RA02195C-s001.pdf]

Electronic Supplementary Material (ESI) for RSC Advances.
This journal is © The Royal Society of Chemistry 2018

Supporting Information

A Sandwich-like Transparent Ceramic Demonstrates Ultraviolet and Visible Broadband Downconversion Luminescence

*Yue Hu, Zheng Li and Wei Pan**

State Key Laboratory of New Ceramics and Fine Processing, School of Materials Science and Engineering, Tsinghua University, Beijing 100084, P. R. China

E-mail: panw@mail.tsinghua.edu.cn

Materials Preparation:

The Ce, Yb: YLZO transparent ceramics were prepared by vacuum sintering with La_2O_3 and ZrO_2 as sintering additives. [1] 99.99% purity commercial powders of CeO_2, Yb_2O_3, La_2O_3, ZrO_2 and Y_2O_3 were weighted and mixed by ball-milling for 16 h. After drying and grinding, the mixed powders were sieved through a 200-mesh nylon screen. The powders were pressed into φ15 mm disks and then cold isostatic pressed at 200 MPa. After sintering in a vacuum furnace at 1800 °C for 20 h under 1.0×10^{-3} Pa, the as-sintered Ce, Yb: YLZO transparent ceramic with brown color was obtained. To obtain the fully-annealed Ce, Yb: YLZO transparent ceramic, dark brown ceramic was annealed at 1450 °C for 10 h in air atmosphere. The sandwich-like Ce, Yb: YLZO transparent ceramic with graded defect structure was prepared by annealling at 1100 °C for 2 h in air atmosphere. For experimental characterization, the the disks were grinded

into uniform size of φ11.80 mm×1.86 mm and were polished on both sides with diamond agent.

Experimental Characterization Details:

The phases of the ceramics were characterized by X-ray diffraction (D/max-2550, Rigaku, Tokyo, Japan) and the morphologies and microstructures were observed by scanning electron microscope (SEM, JSM-7001, JEOL, Tokyo, Japan). The absorption spectra were measured with the UV-VIS-NIR spectrophotometer (UV-3600, Shimadzu, Tokyo, Japan), using a deuterium lamp as light source for the ultraviolet range and a halogen lamp for the visible and near-infrared range. X-ray photoelectron spectroscopy (XPS) of the samples was measured using an X-ray photoelectron spectrometer (Escalab 250Xi, Thermo Fisher Scientifi c, MA, USA). Photoluminescence (PL) and photoluminescence excitation (PLE) spectra were measured with the Fluorescence Spectrometer System (FLS 920, Edinburgh Instruments, Livingston, UK). The detector was Hamamatsu R5509-72 cooled to 80K with liquid nitrogen. The excitation source for regular PL and PLE measurement was a 300W Xenon lamp with monochromator, and the excitation source for PL under solar spectrum was a solar simulator (Microsolar300, Perfect Light Inc., Beijing, China).

First-principle Calculation:

First-principle density functional theory (DFT) was used to calculate the energy band structures of $Ce:Y_2O_3$ and $Ce:Y_2O_{3-x}$. The electronic structure calculations were

performed with density functional theory (DFT) and projector augmented wave (PAW) method as implemented in the Vienna ab initio Simulation Package (VASP). [2, 3] Perdew–Burke–Ernzerh (PBE) function form of generalized gradient approximation (GGA) approach based on plane-wave basis sets was applied in the calculation. [4] In the structural optimization calculation, the energy and force convergence criterion were 10^{-6} eV and 10^{-2} eV, respectively, and the cutoff energy of the plane-wave was set at 500 eV. In the band structure calculation, Gaussian smearing of 0.05 eV and 13 × 13 × 13 k-mesh as well as 440 conduction bands were applied.

Before calculating the band structure, the total free energy of four configurations were calculated. (Fig. S1) In Fig. S1(a), the Ce atom is doped at the 8b site and one adjacent O atom becomes vacancy. The Ce atom in Fig. S1 (b) is doped at same position, but the O vacancy is at second-nearest neighbor to the doped Ce atom. Fig. S1(c) and Fig. S1(d) show the structures with the Ce atom doped at the 24d site and O vacancy at the adjacent and second-nearest position, respectively. From the calculated total free energy we can see that the configuration in Fig. S1(b) is most stable. Therefore we used the structure showed in Fig. S1(b) to calculated the band structure, results are shown in Fig. S2

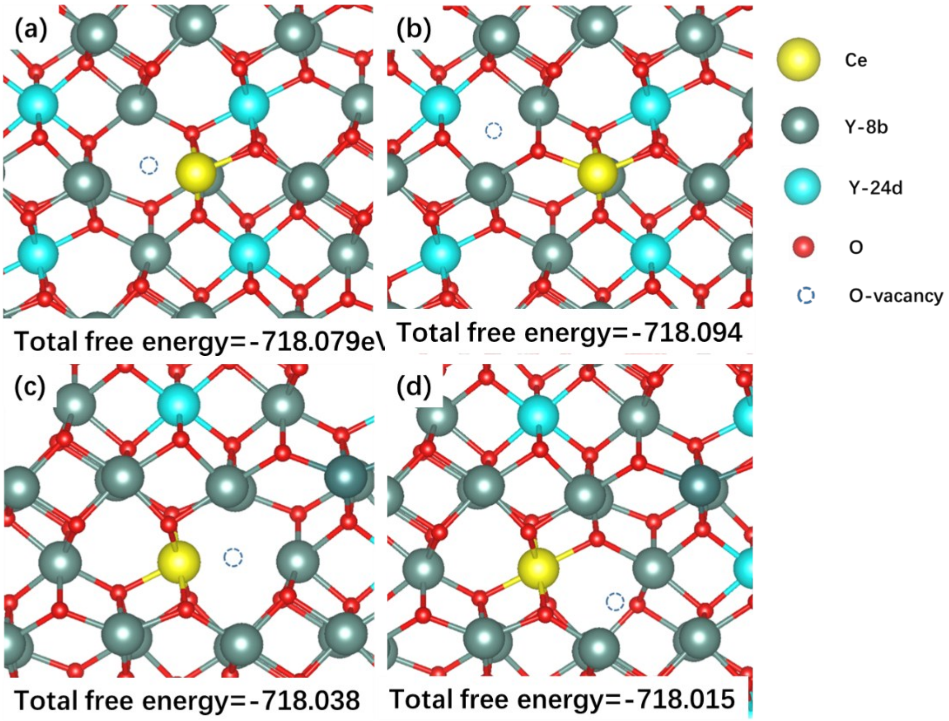

Fig. S1. The total free energy calculated with four kinds of Ce:Y_2O_{3-x} configurations. a) Ce atom doped at the 8b site and oxygen vacancy at the adjacent position. b) Ce atom doped at the 8b site and oxygen vacancy at the second-nearest neighbor position. c) Ce atom doped at the 24d site and oxygen vacancy at the adjacent position. d) Ce atom doped at the 8b site and oxygen vacancy at the second-nearest neighbor position.

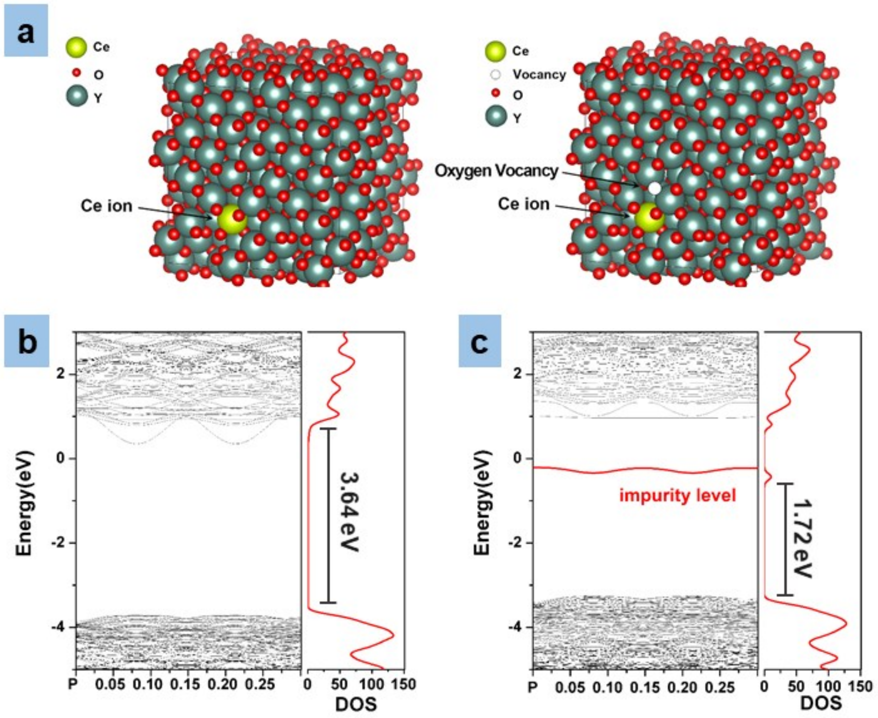

Fig. S2. a) Schematic illustration of the supercell for modelling Ce:Y_2O_3 (left) and Ce:Y_2O_{3-x} (right) with green denoting Y atoms, red denoting O atoms, yellow denoting Ce atoms and open circle denoting the oxygen vacancy (at the most stable position according to the electronic structure calculation); b) Calculated E-V curves and DOS of Ce:Y_2O_3 and Ce:Y_2O_{3-x}.

Reference:

1. L. Zhang, Z. C. Huang and W. Pan, *J. Am. Ceram. Soc.*, 2015, **98**, 824-828.
2. G. Kresse and J. Furthmuller, *Phys. Rev. B*, 1996, **54**, 11169-11186.
3. G. Kresse and J. Hafner, *Phys. Rev. B*, 1993, **47**, 558-561.
4. J. P. Perdew, K. Burke and M. Ernzerhof, *Phys. Rev. Lett.*, 1996, **77**, 3865-3868.
